# Kinetics of Rituximab Excretion into Urine and Peritoneal Fluid in Two Patients with Nephrotic Syndrome

**DOI:** 10.1155/2017/1372859

**Published:** 2017-01-24

**Authors:** Klaus Stahl, Michelle Duong, Anke Schwarz, A. D. Wagner, Hermann Haller, Mario Schiffer, Roland Jacobs

**Affiliations:** ^1^Department of Nephrology, Hannover Medical School, Carl-Neuberg-Strasse 1, 30625 Hannover, Germany; ^2^Department of Hospital Pharmacy, Hannover Medical School, Carl-Neuberg-Strasse 1, 30625 Hannover, Germany; ^3^Department of Clinical Immunology and Rheumatology, Hannover Medical School, Hannover, Germany

## Abstract

Clinical observations suggest that treatment of Rituximab might be less effective in patients with nephrotic range proteinuria when compared to nonnephrotic patients. It is conceivable that the reason for this is that significant amounts of Rituximab might be lost in the urine in a nephrotic patient and that these patients require a repeated or higher dosage. However, this has not been systematically studied. In this case report we describe two different patients with nephrotic range proteinuria receiving Rituximab. The first patient received Rituximab for therapy resistant cryoglobulinemic membranoproliferative glomerulonephritis and the other for second line treatment of Felty's syndrome. We employed flow cytometry to determine the amount of Rituximab excretion in both urine and peritoneal fluid specimens in these patients following administration of Rituximab. We found that a significant amount of Rituximab is lost from the circulation by excretion into the urine. Furthermore we saw a close correlation of the excretion of Rituximab to the excretion of IgG molecules suggesting selectivity of proteinuria as the determining factor of Rituximab excretion. Further larger scale clinical studies could have the potential to evaluate an optimal cut-off value of IgG urinary loss before a possible administration of Rituximab therefore contributing to a more individualized treatment approach in patients with nonselective and nephrotic range proteinuria.

## 1. Introduction

Rituximab is a chimeric monoclonal antibody targeting CD20+ expressing B-cells and is clinically used for a wide range of neoplastic diseases, including indolent and aggressive forms of B-cell non-Hodgkin's lymphoma and B-cell chronic lymphocytic leukaemia, and autoimmune-mediated diseases, such as systemic lupus erythematosus, anti-neutrophil cytoplasmic antibody associated vasculitis, and multiple sclerosis [1–3]. More recently, Rituximab has been also recognized more and more as a second line treatment option in therapy of patients in a wide range of nephrotic diseases, refractory to standard treatment, including steroid dependant and steroid resistant [4–6] or frequent relapsing nephrotic syndrome in children and adults [7, 8], refractory focal and segmental glomerular sclerosis (FSGS) [9], and recurrence of FSGS after renal transplantation [10] as well as membranous nephropathy (MN) [11].

Several clinical observations suggest that treatment with Rituximab in nephrotic patients might be less effective compared to nonnephrotic patients [4, 12]. It is intuitive to speculate that the excretion of 145 kDa Rituximab into the urine due to nonselective large molecular size proteinuria might contribute to this clinical important issue.

Here, we report on two patients with nephrotic range proteinuria receiving Rituximab. We measured Rituximab excretion into the urine and in one case where continuous ambulatory peritoneal dialysis (CAPD) was already initiated in the peritoneal fluid as well, using a flow cytometry based approach. We could demonstrate a significant loss of Rituximab both into the urine and in the peritoneal dialysis fluid.

## 2. Case Presentation

### 2.1. Case One: Rituximab Excretion into the Urine in MPGN Associated Nephrotic Syndrome

#### 2.1.1. History

A 62-year-old male patient was transferred to our renal care unit with a severe case of nephrotic membranoproliferative-glomerulonephritis (MPGN) with mixed cryoglobulinemia. At admission he displayed signs of heavy volume overload, including generalized severe body edema as well as pericardial and pleural effusions. Breathing appeared to be very difficult for the patient and walking or even standing supine was not possible anymore. Severe proteinuria and hypogammaglobulinemia despite regular parenteral immunoglobulin supplementation had caused diverse infectious complications like recurrent pneumonia, erysipelas of both legs, and urinary tract infections. Since we excluded all other possible infectious, autoimmune, and neoplastic differential diagnoses for MPGN and the existing cryoglobulinemia, we attributed his disease to an active but low replicative hepatitis B infection acquired years earlier. The patient had an initial proteinuria of 17450 mg/g creatinine and a serum creatinine of 126 *μ*mol/L corresponding to an estimated glomerular filtration rate (eGFR) of 51 mL/min. eGFR in both patients was calculated using the Chronic Kidney Disease Epidemiology Collaboration (CKD-EPI) equation. The patient showed no signs of peripheral vasculitis or panarteritis nodosa type lesions.

**Figure 1:**
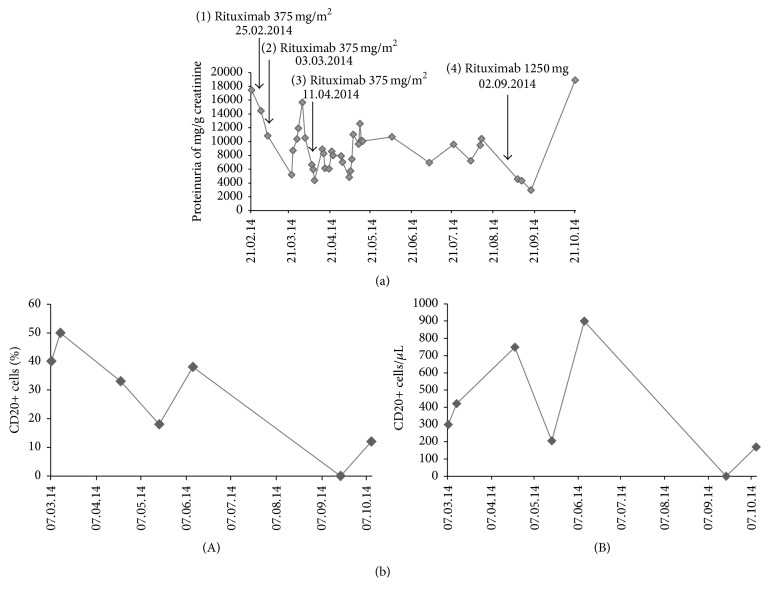
Kinetic of proteinuria (a) and CD20 cell count (b) in the first case. CD20 cell count is both displayed as absolute values (A) as well as percentage (B). Time points of Rituximab administrations are marked accordingly.

The patient was refractory to high doses of steroids. Supportive therapy such as introduction of maximum therapeutic doses of an angiotensin-converting-enzyme inhibitor, aldosterone antagonist, and a high dose diuretic therapy was started and pleural drainage was performed several times. However, the clinical state did not improve nor could the volume overload significantly be reduced by this measures. We therefore decided to administer Rituximab twice in a dose of 375 mg/m^2^ each and seven days apart from each other. The patient additionally received a permanent prophylactic medication of entecavir, which successfully inhibited increased viral replication or a flare of hepatitis under treatment with Rituximab. Proteinuria initially decreased to a minimal of 5206 mg/g creatinine but quickly increased again to the previous treatment value range. The time course of proteinuria is shown in Figure 1(b). Surprisingly, we did not see a substantial suppression of CD20+ B-cells following the first two Rituximab applications, as seen in Figure 1(b). Given the fact that neither proteinuria could be decreased nor did we see a suppression of the target immune cells, we proceeded, about 6 weeks after the first Rituximab administration, to give another dose of 375 mg/m^2^ Rituximab. Again, we only saw a temporal decrease of proteinuria and no substantial suppression of CD20+ B-cell count. Already a few days later, proteinuria rose up again in the high nephrotic range. Unfortunately, the patient developed a severe episode of* Clostridium difficile* associated diarrhea, which leads to sigma colon perforation and required emergency surgery. Postoperative catecholamine dependent sepsis could be managed by broad-spectrum antibiotics and a week of continuous-venovenous-hemodialysis. Although following discharge from intensive care the patient was not dependent on dialysis any more, a cimino fistula was created in prophylactic intention. He made an incomplete recovery, was completely dependent on nursing care, and again severely decompensated with a massive fluid overload and edema. We suspected that a high urinary excretion of Rituximab in this highly nephrotic patient could account for the missing effect of the three preceding Rituximab administrations. Due to the unfavorable prognosis of his disease course we decided following very close informed consent of the patient to administer Rituximab in a higher dose of 1250 mg in total. This time proteinuria indeed decreased to a minimum of 2977 mg/g creatinine and 14 days later CD20+ B-cell count eventually was completely depressed. Unfortunately, he suffered a relapse of peritonitis due to occult perforation. Infection progressed to severe sepsis and because the patient was assessed to be in no adequate condition for repeated surgery, interventional drainage of a paravesical abscess was performed. The patient survived this second severe infectious complication but remained this time dependent on dialysis. Proteinuria and CD20+ B-cell count could not steadily be suppressed and increased again soon after the fourth administration of Rituximab. He refused further treatment and all therapeutic measures including hemodialysis were terminated. The patient was discharged to a palliative care nursery facility and passed away a few weeks later.

#### 2.1.2. Rituximab Kinetics

**Figure 2:**
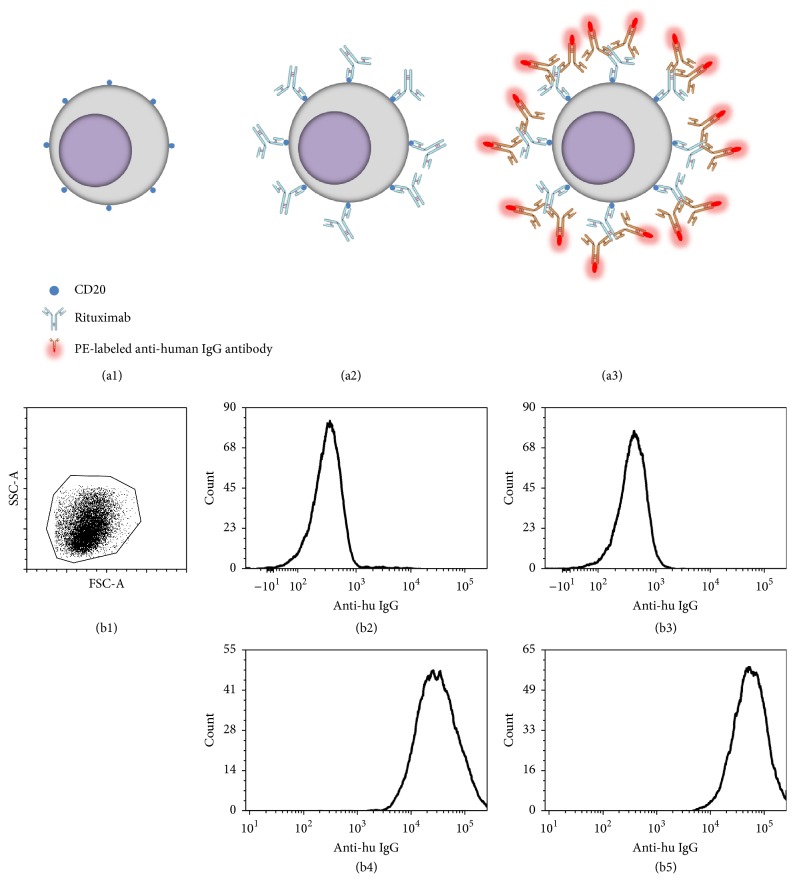
CD20 expressing Daudi cells (a1) were incubated with urine for 20 min to enable suspected Rituximab to bind the CD20 antigen (a2). After three washes in PBS/BSA, cells were incubated with a PE-labeled anti-human IgG antibody and incubated for 20 min (a3). After three washes in PBS/BSA, cells were subjected to flow cytometry analysis. Controls were identically performed but by using Octagam solution instead of urine. Flow cytometric analysis of Daudi cells. Daudi cells were gated according to their FSC/SSC properties (b1). Histograms of Daudi after incubation with PBS (b2), Octagam (b3), urine of the first patient (b4), and peritoneal dialysate fluid of the second patient (b5) are shown. Cell numbers are shown on *y*-axis and relative fluorescence intensity of the secondary (anti-human IgG) antibody on *x*-axis.

The amount of Rituximab excretion into the urine was measured from spot urine samples collected about 24 hours after each of the four Rituximab administrations using flow cytometry. Daudi cells as a CD20 expressing B-cell line were used to determine the Rituximab concentration and Octagam® instead of Rituximab as a negative control. Total IgG levels were determined by nephelometry using a BN ProSpec analyzer (Siemens, Erlangen, Germany). The principle of Rituximab detection by flow cytometry and representative FACS plots are shown in Figure 2. 10000 events of each sample were acquired after gating on Daudi cells according to their FCS/SSC properties. Offline data analyses were performed by using FCS Express V5 and determining mean fluorescence intensity (MFI) of each sample. Values of standard samples with known Rituximab concentration were subjected to statistical analysis in order to calculate nonlinear regression by using Graphpad Prism V6. Based on this calculation MFIs of urine samples were transformed into corresponding Rituximab concentrations.

**Figure 3:**
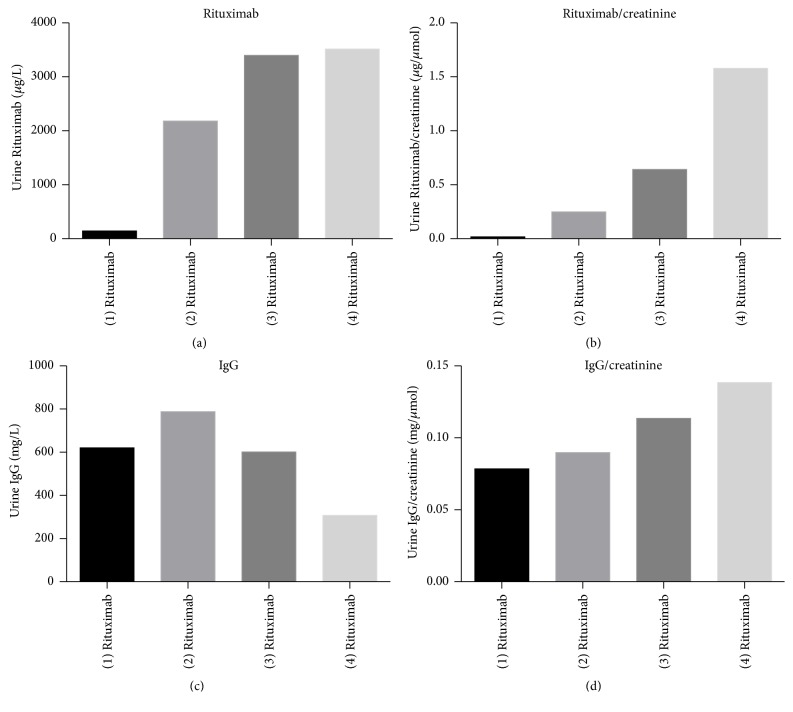
Rituximab urine concentrations (a), urine Rituximab/creatinine ratios as well as IgG urine concentrations (c), and IgG/creatinine ratios in the first case.

Results of Rituximab and IgG measurement in the first case are shown in Figure 3. Since the amount of urinary output and the urine concentration ability of the patient varied significantly between the different probes, we additionally determined urine creatinine and calculated Rituximab/creatinine ratios for all samples.  Figure 3(a) shows Rituximab urine concentrations and Figure 3(b) the corresponding Rituximab/creatinine urine ratios. A significant excretion of Rituximab into the urine is found in all four urine samples. Rituximab urine concentration steadily increases from only 144.67 *μ*g/L after the first Rituximab application up to 3513.57 *μ*g/L after the fourth Rituximab application. The same kinetics are seen when the corresponding Rituximab/creatinine ratios are studied. After the first Rituximab administration the Rituximab/creatinine ratio was only 0.018 *μ*g/*μ*moL, while the ratio increased after the fourth application to 1.57 *μ*g/*μ*moL. This conforms to a 25-fold increase of Rituximab concentration and an 84-fold increase of the Rituximab/creatinine ratio.

We measured Rituximab urinary loss in three patients without proteinuria receiving Rituximab for different indications (two for induction treatment of cANCA associated vasculitis, one for rescue treatment of stiff persons syndrome). In all three control patients no Rituximab could be detected in the patient urine.

Furthermore, to correlate excretion of Rituximab to excretion of IgG molecules, we determined IgG concentration and IgG/creatinine ratios in all samples. Significant amounts of IgG could be detected in all four urine samples. IgG showed varying urine concentrations ranging from 308 mg/L after the fourth up to 788 mg/L after the second Rituximab administration with no clear increasing kinetics. However, when IgG urine excretion was standardized to urine creatinine, an increase of IgG excretion could again be observed ranging from 0.078 mg/*μ*moL after the first up to 0.138 mg/*μ*moL following the last Rituximab administration, which marks an about 2-fold increase. Increasing loss of glomerular selectivity indicated by increased urinary IgG/Creatinine ratio therefore appeared to correlate with increment of Rituximab urinary loss.

### 2.2. Case Two: Rituximab Excretion into the Urine and Peritoneal Dialysate Fluid in IgA-GN Associated Nephrotic Syndrome

#### 2.2.1. History

A 56-year-old male patient presented to our outpatient clinic with chronic symmetric arthralgias of multiple joints, pancytopenia, and a C-reactive protein of 340 U/mL. The patient's blood count was measured as a leukocytopenia of 1.9 Tsd./*μ*L with a neutropenia of 0.7 Tsd./*μ*L, a hemoglobin concentration of 9.2 g/dL, and a thrombocytopenia of 110 Tsd./*μ*L. Rheumatic factor was measured in the normal range but the cyclic citrullinated peptide (CCP) antibody appeared to be very high with 340 U/mL. After infectious, neoplastic, and other rheumatologic differential diagnoses could be ruled out, he was diagnosed with Felty syndrome taking into account the combination of chronic polyarthritis, leukocytopenia, and splenomegaly. Since a combination of colchicum with a course of high dose steroid medication (1 mg/kg equivalent of decortin) did not lead to a clinical remission, it was decided to treat this patient in second line with Rituximab and he was therefore admitted to our renal unit. Additionally, the patient was diagnosed about nine years earlier with IgA-nephropathy with proteinuria in the high nephrotic range. Despite treatment with steroids and cyclophosphamide he eventually progressed to end stage renal disease and started dialysis two years earlier. Because the patient still had a preserved urine output of about 2 l/d it was decided to treat him with continuous ambulatory peritoneal dialysis, which he tolerated well. The patient continued to show proteinuria with 1.95 g/L and a proteinuria to creatinine ratio of 3138 mg/g. He received 500 mg of Rituximab intravenously twice two days apart from each other and tolerated both administrations without experiencing any side effects. CD19/20+ cell count measured about three weeks after Rituximab administration was completely suppressed at 0%. Indeed, arthralgia in all previously affected joint regions subjectively improved significantly about 4 weeks after administration of Rituximab. However, leukocytopenia, anemia, and thrombocytopenia showed no significant signs of recovery until the present time point, which is about 12 weeks after Rituximab administration. Leukocytes were 1.8 Tsd./*μ*L with a neutropenia of 1.0 Tsd./*μ*L, hemoglobin concentration was 9.4 g/dL, and thrombocytes were of 149 Tsd./*μ*L. Proteinuria was unchanged in the nephrotic range with 2.19 g/L and a proteinuria/creatinine ratio of 3800 mg/g.

#### 2.2.2. Rituximab Kinetics

The amount of Rituximab excretion into the urine was measured from 24-hour urine collections, which were started to be collected instantly after each of the two Rituximab administrations. Furthermore, Rituximab concentration was determined from the different bags of effluent of the peritoneal dialysis solution collected after each Rituximab administration. Rituximab was measured in all probes applying FACS technology as described previously.

**Figure 4:**
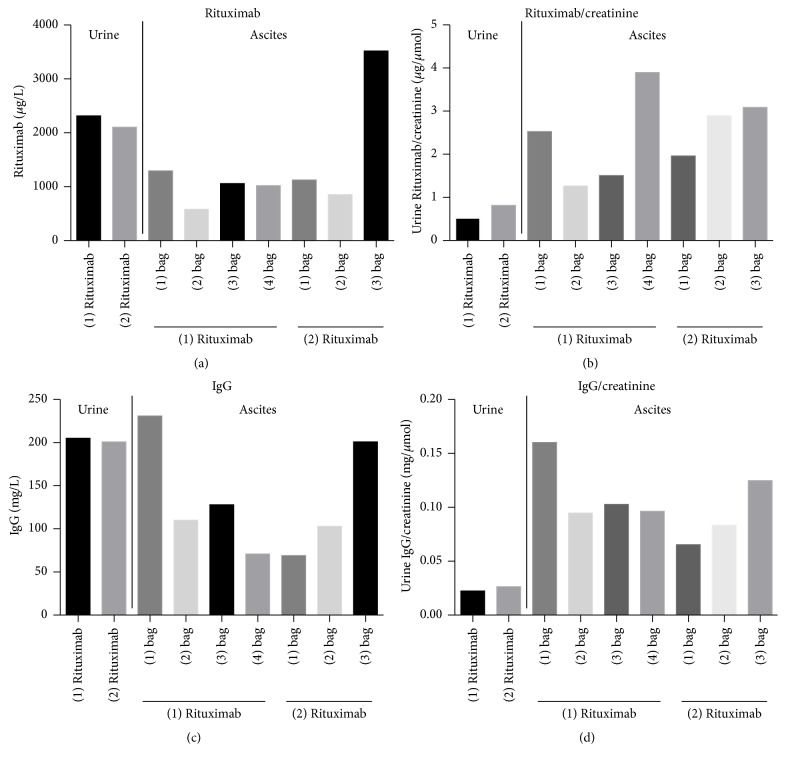
Rituximab urine and peritoneal fluid Rituximab concentrations (a) and Rituximab/creatinine ratios (b), as well as IgG urine and peritoneal fluid concentrations (c), and IgG/creatinine ratios (d) of the second case. Since the second patient performed CAPD with three to four peritoneal fluid bag changes per day, the corresponding effluent bags are numbered in chronological order.

Results are shown in Figure 4. Rituximab was found in high concentrations in both urine and all peritoneal fluid samples that were analyzed. The highest Rituximab excretion into the urine was measured with 2314 *μ*g/L after the first Rituximab administration. The highest excretion into the peritoneal fluid was 3518 *μ*g/L in the third bag after the second Rituximab administration. When Rituximab concentration was standardized to creatinine concentration, Rituximab excretion into the urine and the peritoneal dialysate did increase by time and it showed a slightly additive effect after the second administration.

A significant amount of IgG was found in all urine and peritoneal fluid samples. IgG showed maximum urine concentration of 205 mg/L and a maximum IgG/creatinine ratio of 0.026 mg/*μ*moL, while in the peritoneal fluid a maximum IgG concentration of 231 mg/L could be detected. When IgG/creatinine ratios were obtained, we again saw a correlation of IgG and Rituximab excretion both for urine and peritoneal fluid.

## 3. Discussion

Both patients of this case report received Rituximab not for the reduction of proteinuria as the primary intention. The indication to give Rituximab in the first patient was severe proteinuria resulting from cryoglobulinemia and cryoglobulinemia associated MPGN. Although treatment with Rituximab is well established in hepatitis C associated cryoglobulinemic vasculitis and renal disease [13–16], only case reports exist describing successful utilization of Rituximab in hepatitis B associated cryoglobulinemia [17] and the role of immunosuppression in this disease entity is therefore highly controversial. The second patient received Rituximab as a reserve treatment for Felty syndrome. Successful treatment of Felty syndrome associated neutropenia and synovitis has been described in a series of different case reports [18–20].

Counsilman et al. recently described a case of one pediatric patient with steroid resistant nephrotic syndrome and biopsy proven minimal change nephropathy, who received Rituximab as second line treatment [21]. The authors used a direct enzyme linked immunosorbent assay to detect high levels of Rituximab in the patients urine and pleural fluid and calculated, following a two-compartment pharmacokinetic model [22], urinary clearance of Rituximab as about 25% of the total clearance and a very short serum half-life of less than one day compared to about 20 days in nonnephrotic patients [21]. While the previous studies used ELISA technology to detect Rituximab, we employed a novel flow cytometry based approach using Daudi cells as a CD20 expressing B-cell line to measure Rituximab concentrations. Another small size case series of pediatric patients with steroid dependant nephrotic syndrome reported a smaller reduction of median serum half-life of about 15 days [23].

A significant amount of Rituximab excretion into the urine could be detected in both patients. While Counsilman et al. reported a maximum excretion of Rituximab into the urine of about 12000 *μ*g/L at a very high proteinuria of 19469 mg albumin/g creatinine, the first patient had a maximum excretion of Rituximab of 3513 *μ*g/L at a proteinuria of 10662 mg/g creatinine, while in the second patient's urine a maximum Rituximab concentration of 2314 *μ*g/L was measured at a proteinuria of 3138 mg/g creatinine. This suggests that the amount of Rituximab excretion into the urine roughly correlates with the degree of proteinuria in these three patients despite different etiologies of the present nephrotic syndromes (steroid resistant nephrotic syndrome, MPGN, and IgA-nephropathy) and despite the different demographic patient characteristics (pediatric versus adult patients). However, due to a great degree of variation in the degree of proteinuria in the first patient (see Figure 1(a)) and since not always proteinuria values have been obtained at the very day of Rituximab administration in this patient, it is not possible to draw a precise conclusion concerning a direct correlation.

We hypothesized that the amount of Rituximab loss into the urine might be largely dependent on the degree of selectivity loss of the glomerular filter. Therefore, we additionally measured IgG excretion in all acquired samples and indeed a substantial amount of IgG secretion was found in all patient urine samples. When Rituximab and IgG urine levels were standardized to the creatinine concentration, we could see a very close correlation of Rituximab/creatinine with the IgG/creatinine ratios. Thus when IgG urine secretion increased, so did Rituximab loss into the urine. Interestingly, the first patient showed clearly a stepwise increasing Rituximab excretion into the urine, despite that his level of proteinuria greatly varied over time (and even was greatest before the first Rituximab administration) and despite that the Rituximab administrations were partly separated a few weeks from each other, which excludes considering the severely reduced serum half-time of less than a day and additive effects. Since IgG excretion into the urine also increased in a stepwise fashion in this patient's case, we speculate that progressive loss of glomerular selectivity may be responsible for this.

In the first case we saw an incomplete B-cell depletion despite the first three Rituximab administrations and a complete B-cell depletion with the higher dose of 1250 mg of Rituximab. Since we measured B-cell count always about 10–14 days after Rituximab administration we cannot exclude temporal complete B-cell depletion followed by a fast rebound due to a severely reduced Rituximab serum half-time as described by Counsilman et al. [21]. High Rituximab excretion into the urine might on the other hand prevent complete CD20+ cell depletion therefore contributing to poor treatment response.

Excretion of Rituximab into the pleural fluid in nephrotic syndrome has been described previously [21]. Interestingly we found in the second case a substantial excretion of Rituximab into the peritoneal fluid. Maximum Rituximab excretion into the peritoneal fluid was with 3518 *μ*g/L significantly higher than the previously reported maximal secretion of Rituximab into the pleural fluid with about 700 *μ*g/L [21]. Surprisingly, when Rituximab and IgG levels in the peritoneal fluid samples were standardized to the corresponding creatinine concentration, again a clear correlation of IgG and Rituximab excretion could be seen. Since this patient received two doses of Rituximab only two days apart, excretion of Rituximab in the peritoneal fluid, when expressed as creatinine ratios, additionally exhibited a time dependent and slightly additive pattern. Rapid loss of Rituximab into the urine and in the third space compartments might have contributed again to poor treatment success in this patient.

To our knowledge, this is the first report describing Rituximab kinetics in two adult nephrotic patients, in one patient, who demonstrates no adequate suppression of CD20+ B-cells despite two applications of Rituximab in short distance from each other, and in the other patient showing excretion of Rituximab in both urine and peritoneal dialysate fluid. We used a novel approach to measure Rituximab applying flow cytometry based detection on a CD20+ cell line. Our data demonstrate a possible close correlation of Rituximab excretion into urine and peritoneal fluid with the amount of IgG secretion suggesting a loss of filter selectivity as the primary determinant of Rituximab urine excretion. The first case shows that high Rituximab excretion into the urine might prevent complete CD20+ cell depletion, therefore contributing to poor treatment response. The second case demonstrates that Rituximab can be lost not only into the urine but also in the third space again causing reduced treatment efficacy.

Future larger scale clinical studies will have to confirm that high nonselective protein loss significantly correlates with an increased excretion of Rituximab into the urine and poor treatment response rate in nephrotic diseases. Evaluating an optimal cut-off value of IgG urinary loss before a possible administration of Rituximab could significantly contribute to a patient individualized treatment approach in refractory nephrotic syndrome.
